# Coupling mechanism between wear and oxidation processes of 304 stainless steel in hydrogen peroxide environments

**DOI:** 10.1038/s41598-017-02530-5

**Published:** 2017-05-24

**Authors:** Conglin Dong, Chengqing Yuan, Xiuqin Bai, Jian Li, Honglin Qin, Xinping Yan

**Affiliations:** 1 School of Energy and Power Engineering, Wuhan University of Technology, Wuhan, 430063 China; 2 State Key Laboratory of Tribology, Tsinghua University, Beijing, 100084 China; 3 Wuhan Research Institute of Materials Protection, Wuhan, 430030 China; 4 College of Mechanical and Power Engineering, China Three Gorges University, Yichang, 443002 China

## Abstract

Stainless steel is widely used in strongly oxidizing hydrogen peroxide (H_2_O_2_) environments. It is crucial to study its wear behaviour and failure mode. The tribological properties and oxidation of 304 stainless steel were investigated using a MMW-1 tribo-tester with a three-electrode setup in H_2_O_2_ solutions with different concentrations. Corrosion current densities (CCDs), coefficients of frictions (COFs), wear mass losses, wear surface topographies, and metal oxide films were analysed and compared. The results show that the wear process and oxidation process interacted significantly with each other. Increasing the concentration of H_2_O_2_ or the oxidation time was useful to form a layer of integrated, homogeneous, compact and thick metal oxide film. The dense metal oxide films with higher mechanical strengths improved the wear process and also reduced the oxidation reaction. The wear process removed the metal oxide films to increase the oxidation reaction. Theoretical data is provided for the rational design and application of friction pairs in oxidation corrosion conditions.

## Introduction

High-concentration hydrogen peroxide (H_2_O_2_) is an ideal green propellant because it is non-toxic, non-polluting, and easy to store and has a high density and a high specific heat^1–4^. In liquid propellant rocket engines of new-generation, non-toxic, harmless launch vehicles, some moving parts, such as turbopump bearings, sealing parts, and valves, must be operated in a H_2_O_2_ solution, which is strongly oxidative^5–9^. The operation time of moving parts may not be very long, but their wear surfaces are still easily oxidized because of the strong oxidation, and the tribological properties and mechanical properties change significantly. Thus, the functional implementation and working reliability of the propulsion system will be restricted^10–15^. To achieve stable and long-term operation of hydrogen peroxide propulsion systems, it is urgent to develop compatible and wear-resistant tribo-pair materials serviced in H_2_O_2_ solutions. Therefore, it is very important to choose reasonable rubbing pairs that can operate in H_2_O_2_ solutions. 304 stainless steel possesses not only good mechanical strength and processing properties but also intercrystalline corrosion resistance. As a new material, Si_3_N_4_ ceramics have high-temperature resistance, high hardness, wear resistance, corrosion resistance, and low density. Therefore, they are widely used in H_2_O_2_ solutions in aerospace applications^16–19^.

To reveal the oxidation reaction mechanisms of the stainless steel in the strong oxidation environment, Gao investigated the microstructure and chemical compositions of oxide films formed on surfaces of stainless steel in the H_2_O_2_ solutions. He found that a duplex-layer structure was identified in the oxide films. The loose outer layer was rich in Fe, while the compact inner layer was rich in Cr and Fe. In addition, Ni enrichment was observed at the interface between the metal matrix and oxides^20^. Maryam studied the oxidation products using aqueous suspensions of metal powder in the H_2_O_2_, and found that corresponding activation energies at room temperature were 52 ± 4, 44 ± 5 and 57 ± 7 kJ mol^−1^ for Fe_3_O_4_, Fe_2_CoO_4_ and Fe_2_NiO_4_, respectively^21^. Yeh explored the static corrosion current density of the reduction reaction for 304 stainless steels treated using zirconium oxide in the hydrogen peroxide^22^. Yu and Dong reported the tribological properties of the high entropy alloys under hydrogen peroxide solutions^23, 24^, and found that the tribological behaviors depended on the counterparts and structure of high entropy alloys. However, the relationships between the tribological behaviour and dynamic oxidation process of wear components, those are operated in the strongly oxidizing environment of H_2_O_2_ solutions, remain an open research topic. This project aims to determine the coupling mechanism between the wear and oxidation processes of the 304 stainless steel ring-disc in H_2_O_2_ solutions of different concentrations.

## Results

### Analysis of CCDs

**Figure 1:**
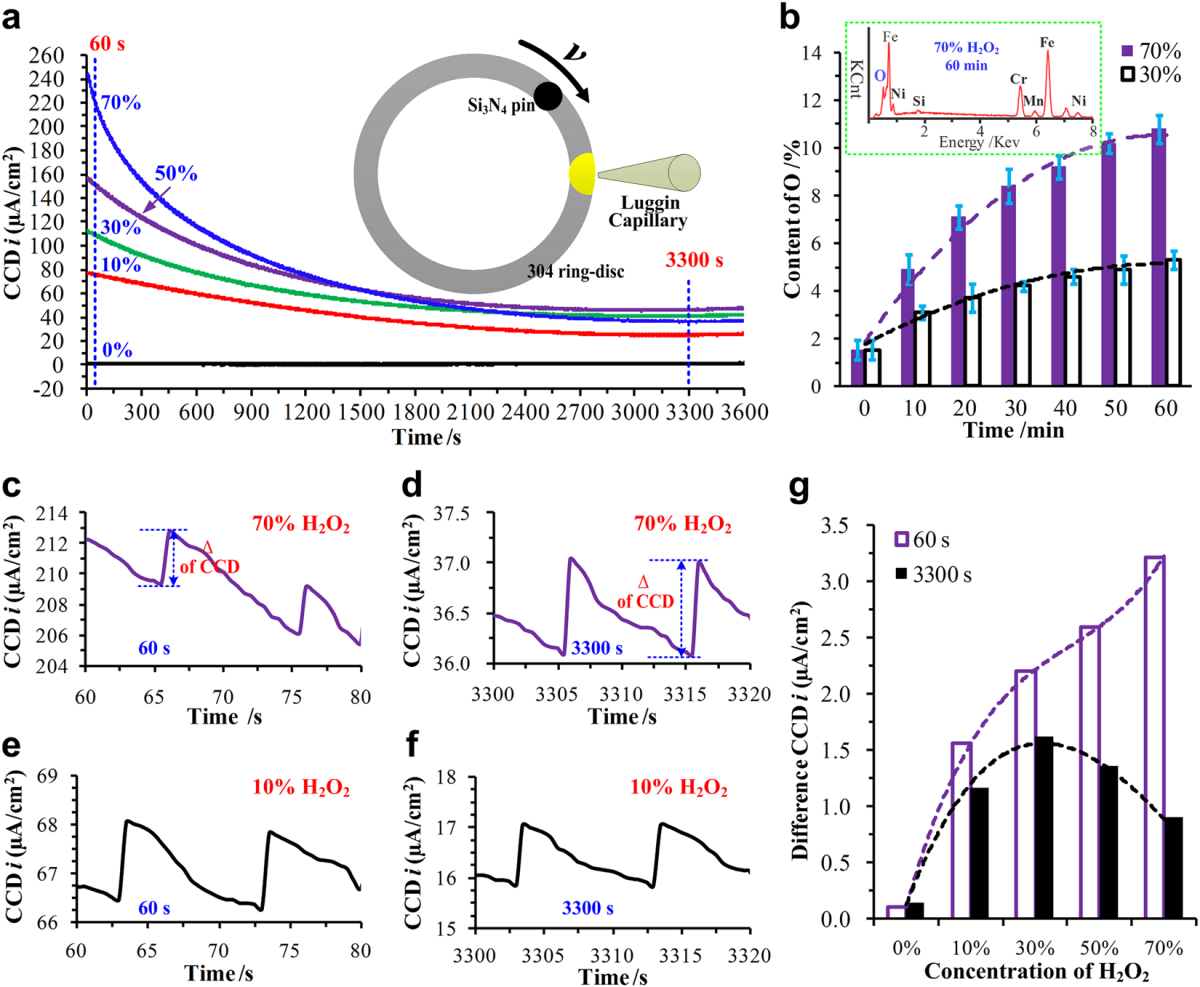
Behaviours of the CCDs. (**a**) Trend of the CCDs of the 304 ring-discs in different H_2_O_2_ solutions, (**b**) the average oxygen contents of the wear surfaces subjected different oxidation times with 70% and 30% H_2_O_2_ solutions, CCDs in the 70% H_2_O_2_ solution at (**c**) 60 s and (**d**) 3300 s, CCDs in the 10% H_2_O_2_ solution at (**e**) 60 s and (**f**) 3300 s, and (**g**) increasing amplitudes of the CCDs for one cycle in different H_2_O_2_ solutions at 60 s and 3300 s.

As was the working electrode in the three-electrode setup as shown in Fig. S1, 304 stainless steel was corroded in the H_2_O_2_ solution. In fact, the corrosion mechanism was the oxidation reaction between the easily oxidized metal elements and H_2_O_2_ solution. The current was formed between the working electrode and the auxiliary electrode. The current density was measured and named as corrosion current density (CCD). The CCD can quantitatively characterise the degree of the oxidation reaction of the 304 stainless steel, and the higher the CCD, the stronger the oxidation reaction^25^. Figure 1 shows the CCDs of the 304 ring-discs in different concentrations of H_2_O_2_. The CCD in the 0% H_2_O_2_ solution was almost equal to zero and was the lowest. The CCDs in the other H_2_O_2_ solutions were much higher and revealed that the ring-discs were significantly oxidized. At the beginning, the CCDs were at high levels, and the greater the concentration of the H_2_O_2_ solution, the higher the initial CCD. These results suggest that the higher the concentration, the stronger the oxidation reaction of the H_2_O_2_ solution. With increasing test time, the CCD decreased and became stable because the wear surfaces of the ring-discs were coated with the remnant metal oxide products, even though the ceramic pins slid on the wear surfaces and removed some of the metal oxide products. These phenomena can be proved by the test data shown in Fig. 1b, which presents the average oxygen contents of the wear surfaces with different oxidation times in the 70% and 30% H_2_O_2_ solutions. The average oxygen contents increased as the oxidation time increased, and the slope of the curve decreased. Moreover, the average oxygen contents in the 70% H_2_O_2_ solution were much higher than those in the 30% H_2_O_2_ solution, and were especially obvious after oxidizing for a long time. Those phenomena reflected that more metal oxide products remained on the wear surfaces in the high concentration of H_2_O_2_ solutions. The metal oxide products prevented the fresh metal contacting the H_2_O_2_ solution, and reduced the further oxidation reaction^26^. Therefore, the CCD in the 70% H_2_O_2_ solution decreased sharply as the test time increased and was lower than those in the 30% and 50% H_2_O_2_ solutions when the test time exceeded 40 min as shown in Fig. 1a.

Figure 1c–f shows the details of the CCDs in the 70% and 10% H_2_O_2_ solutions at 60 s and 3300 s. The CCDs were nonstationary and showed a zigzag fluctuation because the ceramic pins slid on the wear surfaces periodically. The CCDs reflected that the wear processes had a significant influence on the oxidation processes. When the ceramic pins passed through the wear surfaces, some of the metal oxide products were removed, and fresh metal was exposed and put in contact with the H_2_O_2_ solution, which resulted in increased oxidation reaction and CCDs. Obviously, the greater the amount of fresh metal in contact with the H_2_O_2_ solution, the greater the increasing amplitude of the CCDs. Figure 1g shows the increasing amplitudes of the CCD for one cycle in different H_2_O_2_ solutions at 60 s and 3300 s. Generally, the increasing amplitudes of the CCDs at the beginning (60 s) were higher than those later in the wear process (3300 s) in H_2_O_2_ solutions with the same concentration. The test data demonstrated that more new metal material was exposed to the H_2_O_2_ solution in one cycle at the beginning compared to the later period. Their differences increased sharply increasing H_2_O_2_ concentration. At the beginning, the increasing amplitudes of the CCD increased as the concentration increased. Later in the wear process, the increasing amplitudes of the CCD in the 30% H_2_O_2_ solution were the greatest and decreased as the concentration increased. What’s more, the increasing amplitude of the CCD in the 70% H_2_O_2_ solution was smaller than those in the 10% H_2_O_2_ solution. Obviously, more metal oxide products remained and adhered to the wear surfaces of the 304 ring-discs in later period and at high concentrations of H_2_O_2_ (50% and 70%), and weakened the oxidation reaction, which were proved by the data in Fig. 1b indirectly.

### Analysis of wear mass losses

**Figure 2:**
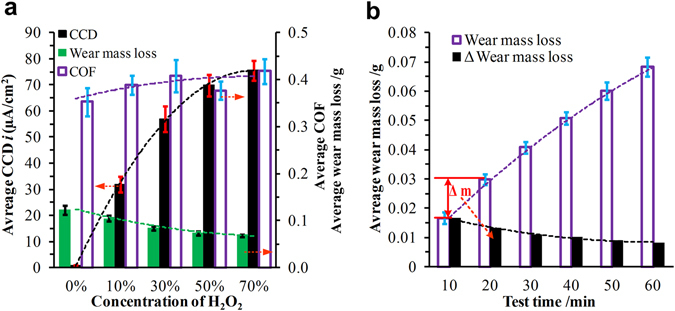
(**a**) Average wear mass losses of the ring-discs, COFs, and CCDs in different H_2_O_2_ solutions and (**b**) average wear mass losses during different oxidation processes in the 70% H_2_O_2_ solution.

Figure 2a presents the average wear mass losses of the ring-discs, COFs, and CCDs in the H_2_O_2_ solutions. The average COF subjected slight effects as the concentration of H_2_O_2_ increased. The average CCDs in H_2_O_2_ solutions (10%, 30%, 50%, and 70%) were much higher than that in pure water. They increased significantly with increasing H_2_O_2_ concentration and increased slowly in 70% H_2_O_2_ solutions. However, the average wear mass losses showed a tendency opposite that of the average CCDs and decreased as the concentration of H_2_O_2_ increased, and the decreasing trend became less steep with high concentrations of H_2_O_2_. Thus, high concentrations of H_2_O_2_ enhanced the wear resistance of 304 ring-discs. Figure 2b shows the wear mass losses during different oxidation times with 70% H_2_O_2_ solution. Generally, the wear mass loss increased as the test time increased, but the slope decreased. The change of the wear mass losses between the two wear mass losses with adjacent sliding times (every 10 min) were obtained, which revealed the effect of the oxidation processes on the wear mass losses. The wear mass loss clearly decreased after oxidizing for a longer time. It is reasonable to infer that the different degrees of oxidation during the wear test influenced on the metal oxide films, and had a great effect on the wear resistance of the 304 ring-disc.

### Analysis of surface topographies

**Figure 3:**
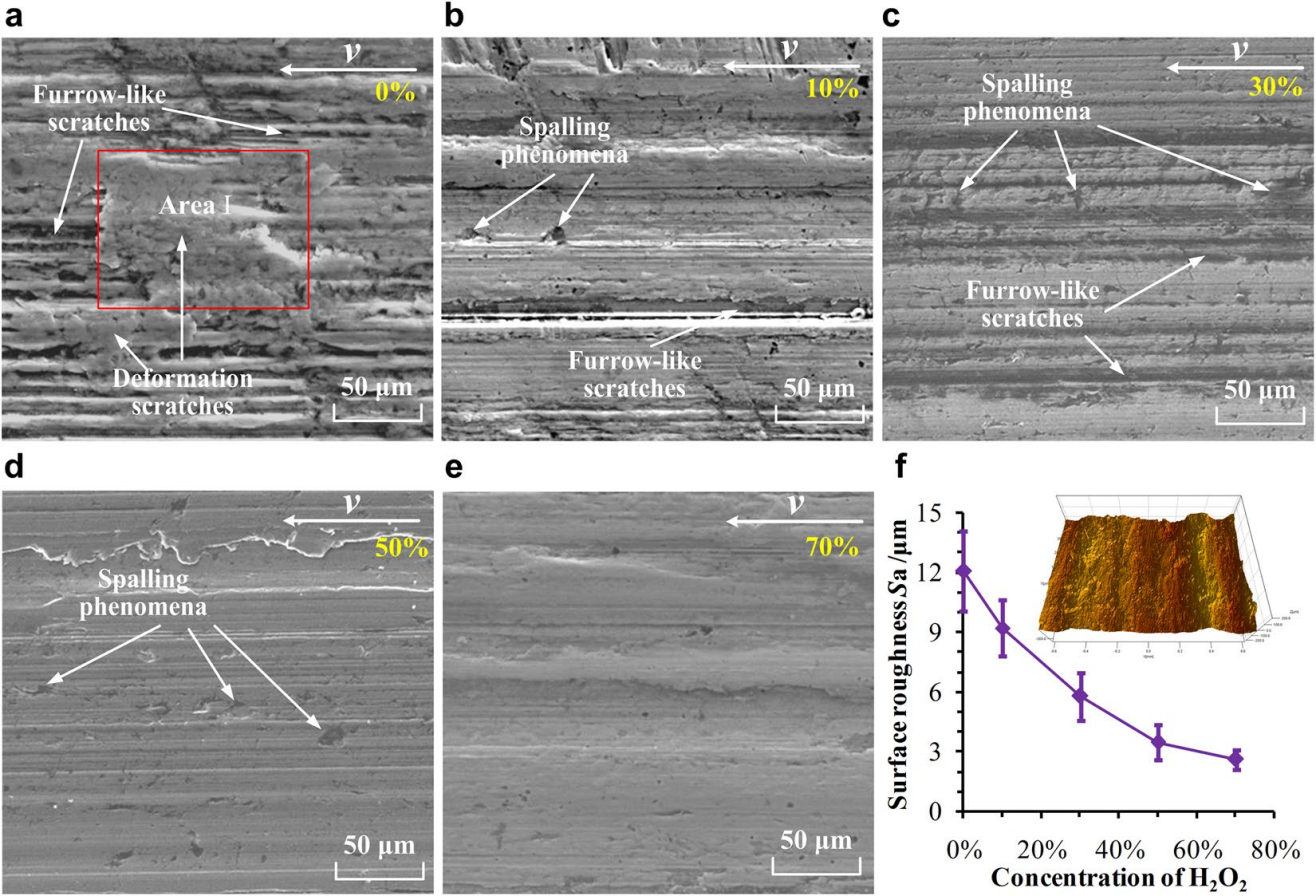
SEM images of wear surfaces of the 304 ring-discs tested in (**a**) 0%, (**b**) 10%, (**c**) 30%, (**d**) 50%, and (**e**) 70% H_2_O_2_ solutions and (**f**) surface roughness of the wear surfaces.

For further insight into the wear process, the surface topographies and surface roughness of the wear surfaces of the 304 ring-discs were examined using scanning electron microscopy (SEM) and white-light interference microscopy, respectively, and the results are shown in Fig. 3. Figure 3a shows that there were not only many significant, deep, wide furrow-like scratches but also deformation scratches accumulated with the plastic deformation materials distributed on the wear surface of the ring-disc tested in pure water (0%). The deformation materials (see area I in Fig. 3a) were rich in metal elements of Fe, Cr, Mn and Ni as shown in the elemental analysis result in Fig. S2, and were proved to be the stainless steel. In the 10% and 30% H_2_O_2_ solutions, the furrow-like scratches were significantly reduced, and there were no obvious deformation scratches, but spalling occurred. When the concentration of the H_2_O_2_ solution was 50% or 70%, there were no obvious furrow-like or deformation scratches on the wear surfaces, and spalling was also clearly reduced. The characteristics revealed that high concentrations of H_2_O_2_ effectively reduced the abrasive wear and deformation scratches and led to smooth wear surfaces of the ring-discs, similar to polishing^27^. Figure 3f shows that the *S*_*a*_ decreased sharply as the concentration of H_2_O_2_ increased. The numerical data were consistent with the results of visual inspection presented in Fig. 3a–e. The observed features depend the metal oxide films that covered on the wear surfaces^28^.

### Analysis of metal oxide films

**Figure 4:**
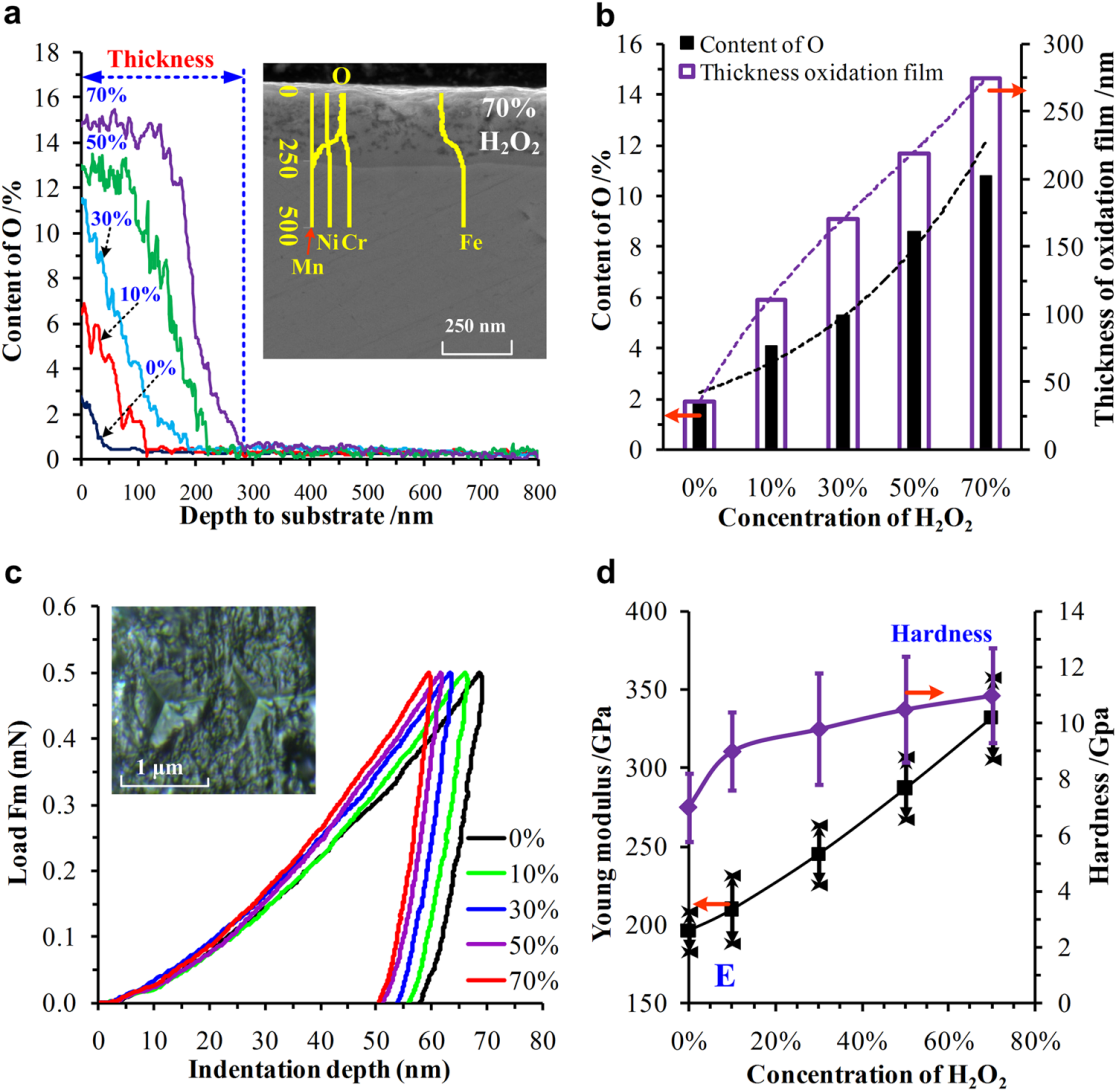
Characteristics of the metal oxide films covering the wear surfaces of the ring-discs. (**a**,**b**) Thicknesses and oxygen contents of the metal oxide films and (**c**,**d**) Young modulus and hardness of the metal oxide films at room temperature.

Because there are large differences in the friction and oxidation behaviours in H_2_O_2_ solutions, the metal oxide films covering the wear surfaces of the ring-discs have played an important role in this boundary lubrication state. The thicknesses, oxygen contents, Young moduli, and hardnesses of the metal oxide films were characterized, as shown in Fig. 4. Generally, the oxygen contents decreased as the depth from the surface increased, as shown in Fig. 4a. The oxygen contents in H_2_O_2_ solutions (10%, 30%, 50% and 70%) were much higher than that in pure water (0%). In the 50% and 70% H_2_O_2_ solutions, the oxygen contents remained steady at depths of 95 nm and 162 nm, respectively. These results demonstrate that the ring-discs were oxidized adequately under high concentrations of H_2_O_2_. The average oxygen contents and thicknesses of the metal oxide films increased as the concentration of H_2_O_2_ increased, as shown in Fig. 4b. Therefore, it is reasonable to assume that the metal oxide film became more complete, uniform, and compact under high concentrations of H_2_O_2_ or after oxidizing for a long time. Figure 4c,d displays the results of the Young modulus and hardness of the metal oxide films characterized by nano-indentation at room temperature. The Young modulus and hardness increased as the concentration of H_2_O_2_ increased significantly, and reflected that metal oxide films had a higher Young’s modulus and hardness than the original steel^29, 30^. The higher mechanical strengths of the metal oxide film can explain the better anti-wear capability of the ring-discs, which decreased the wear mass losses, as shown in Fig. 2. Many researchers obtained similar results^31, 32^.

### Analysis of compositions

**Figure 5:**
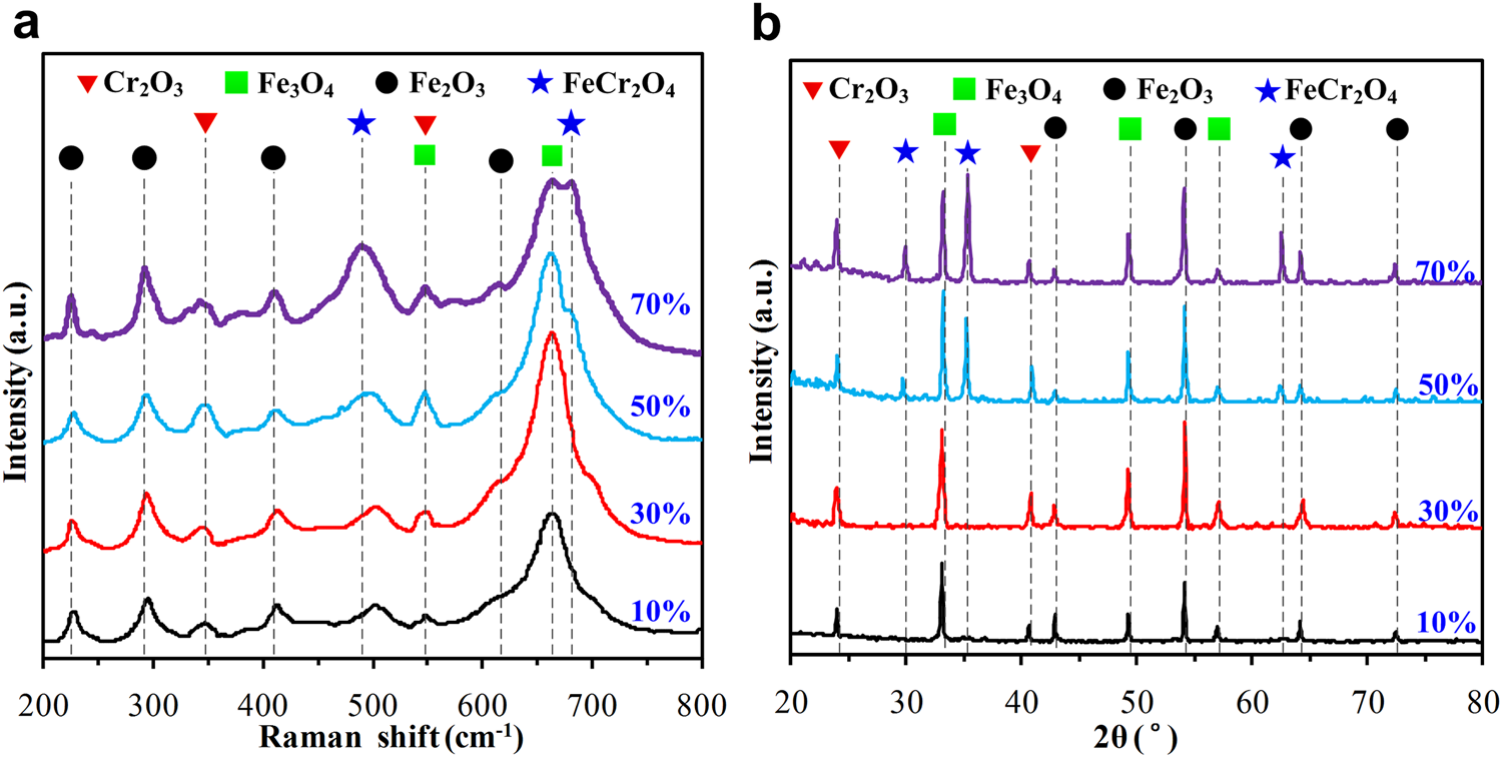
Compositions of the metal oxide films in 10%, 30%, 50% and 70% H_2_O_2_ solutions were measured using (**a**) Raman spectroscopy and (**b**) XRD.

To further investigate the phase structure, the metal oxide films produced in 10%, 30%, 50%, and 70% H_2_O_2_ solutions were measured using Raman spectroscopy and XRD, as shown in Fig. 5. With 10% and 30% H_2_O_2_ solutions, the metal oxide films were mainly Fe_2_O_3_, which has characteristic peaks around 224.5, 290.5, 412.5, and 613.5 cm^−1^, Fe_3_O_4_ (543.5 and 669.5 cm^−1^) and Cr_2_O_3_ (347 and 548 cm^−1^)^33^, as shown in Fig. 5a. The characteristic peaks of Fe_2_O_3_, Fe_3_O_4_, and Cr_2_O_3_ for films produced in the 50% and 70% H_2_O_2_ solutions were higher, especially in 70% H_2_O_2_ solution. In addition, FeCr_2_O_4_, which was represented by the characteristic peaks around 486.5 and 702 cm^−1^, was present^34^. These test data suggest that the oxidation became more severe as the concentration of H_2_O_2_ increased and more metal was oxidized. The numerical data were consistent with the results presented in Fig. 4a and b. Previous reports showed that Cr_2_O_3_ and FeCr_2_O_4_ prevented the oxidation of the metal on the wear surface by the H_2_O_2_ solution and further enhanced the mechanical strength of the metal oxide film^35, 36^. The XRD analysis presented characteristic peaks of Fe_2_O_3_ (2θ around 43°, 54°, 64.4°, and 72.5°), Fe_3_O_4_ (2θ around 32.2°, 49.3°, and 57.1°), and Cr_2_O_3_ (2θ around 24° and 40.9°) in the 10% and 30% H_2_O_2_ solutions^37, 38^. However, in addition Fe_2_O_3_, Fe_3_O_4_, and Cr_2_O_3_, characteristic peaks around 2θ = 30°, 35.3°, and 62.5°, indicating FeCr_2_O_4_, also occurred^39, 40^. Thus, the Raman analysis and XRD analysis showed similar results.

## Discussion

### Effects of concentrations of H_2_O_2_ on wear and oxidation processes

The metal oxide film that formed in H_2_O_2_ solution had a significant influence on the wear and oxidation processes. In pure water, the oxidation reaction was too weak to form a metal oxide film. Thus, the abrasive wear between the 304 ring-disc and Si_3_N_4_ ceramic pin rubbing pairs was serious, as demonstrated by the wear features shown in Fig. 3a. As a result, the wear mass loss and *S*_*a*_ were greater than those with the H_2_O_2_ solutions.

As is well known, 304 stainless steel includes Fe, Cr, Ni, and other metal elements. In low concentrations of H_2_O_2_ (10% and 30%), the metal elements on the wear surfaces of the ring-discs were oxidized into Fe_2_O_3_, Fe_3_O_4_, and Cr_2_O_3_ the during wear process, as shown in Fig. 5. The metal oxidation products covered the wear surfaces and formed a layer of metal oxide film. They prevented the exposure of fresh metal to the H_2_O_2_ solutions, which reduced the oxidation reaction and CCDs. Moreover, metal oxide films with higher Young moduli and hardnesses were beneficial to reduce the abrasive wear and thus enhance the wear resistance, and eventually reduced the mass losses compared with those in pure water, as presented in Figs 2a and 4c,d.

The oxidation reaction in the high-concentration H_2_O_2_ solutions (50% and 70%) was much stronger than that in the low-concentration H_2_O_2_ solutions, which was demonstrated by the data in Figs 1a,b, 2a and 4a,b. In addition to Fe_2_O_3_, Fe_3_O_4_, and Cr_2_O_3_, FeCr_2_O_4_ was found in the oxidative products (Fig. 5). The wear surfaces of the ring-discs were oxidized quickly, and integrated, homogeneous, and compact metal oxide films were formed and became thicker. Their Young modulus and hardness values were further increased, as shown in Fig. 4c,d. The abrasive wear was reduced further. Both the wear mass loss and surface roughness were the smallest, as shown in Figs 2a and 3f. Furthermore, the CCD decreased significantly because of the thick and compact metal oxide film.

### Effects of oxidation time on wear and oxidation processes

At the beginning, the metal oxide films were too thin and incomplete to be easily removed, and, consequently, the CCDs and its increasing amplitudes were at high levels as shown in Fig. 1a and g. With increase in the wear and oxidation process, more metal oxidative products accumulated on the wear surfaces, which proved by the data in Fig. 1b. The metal oxide films became thick, integrated and hard, which resulted in prevented the oxidation reaction further. Moreover, the wear mass losses were reduced as shown in Fig. 2b. Thus, the fresh metal, which emerged in the H_2_O_2_ solutions after the Si_3_N_4_ ceramic pin passed through the wear surfaces for one cycle, was also reduced. As a result, the CCDs and their increasing amplitudes were smaller than those at the beginning as shown in Fig. 1g. The test data reflected that increasing the oxidation time was useful to reduce the wear mass loss and oxidation reaction.

### Coupling mechanism between wear and oxidation processes

**Figure 6:**
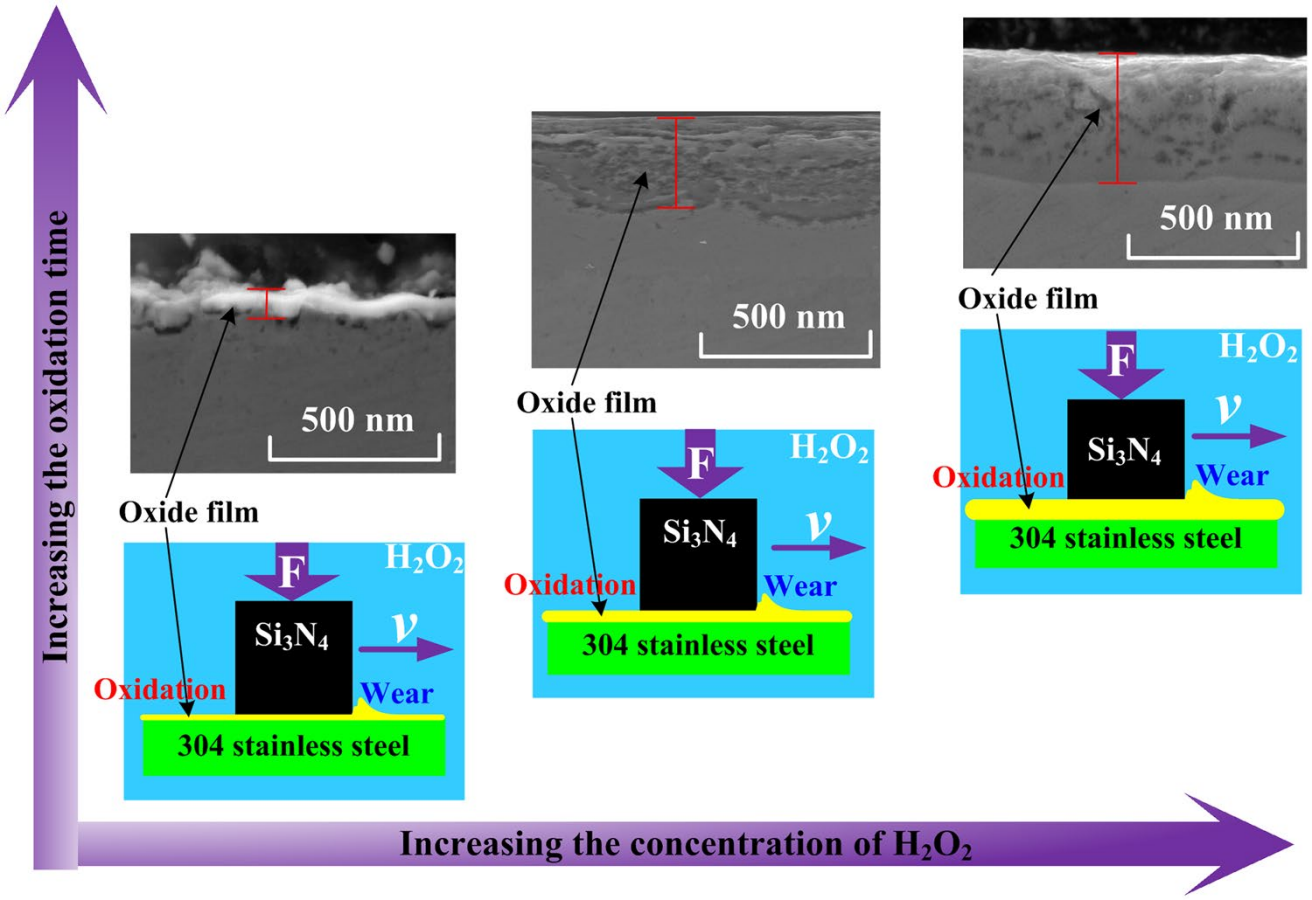
The coupling mechanism between the wear and oxidation processes.

All of the test results show that the wear process and oxidation process interact with each other in the H_2_O_2_ solutions. The higher mechanical strengths of tribo-films are helpful for the anti-wear performance. Figure 6 presents the coupling mechanism between them for the 304 stainless steel ring-disc in the H_2_O_2_ solutions. The easily oxidation elements on the worn surfaces of the ring-discs are strongly and quickly oxidized by H_2_O_2_ to form a layer of metal oxide film. The metal oxide film can prevent contact between the metal elements within the ring disc and the H_2_O_2_ solution and reduce the oxidation reaction effectively. At the same time, friction and wear destroyed and removed the metal oxide films, which caused the film to be thin or discontinuous, which resulted in an increase in the oxidation reaction. However, the metal oxide films could not be removed completely. With an increase in the concentration of H_2_O_2_ or the oxidation time, more and more metal oxide products accumulated on the wear surface and formed a layer of integrated, homogeneous, and compact metal oxide film. With greater Young modulus and hardness, the metal oxide film could reduce the abrasive wear to improve the wear process and also further reduce the oxidation reaction.

## Conclusion

The wear and oxidation processes of 304 stainless steel were investigated under different concentrations of H_2_O_2_ solution. The results show that the wear and oxidation processes interacted with each other. Metal oxide films with higher mechanical strengths formed during the wear tests in H_2_O_2_ solutions and reduced the abrasive wear, which reduced the surface roughness, enhanced the wear resistance, and also further reduced the oxidation reaction. With an increase in the concentration of H_2_O_2_ or the oxidation time, the denser, compact, thick and hard the metal oxide film became, and the greater its influence became. However, the wear process removed the metal oxide films, which increased the oxidation reaction. The knowledge gained in the present work will be useful for the rational design and application of friction pairs in oxidation corrosion conditions.

## Methods and Experiments

### Materials preparation

The inner diameter, outer diameter, and thickness of the 304 stainless steel ring-discs were 56, 64, and 8 mm, respectively. Their wear surfaces were polished with grit polishing paper to remove the oxide layer, and they were placed in a vacuum environment before testing. Their mean surface roughness (*S*_*a*_) were 0.2 ± 0.05 μm. The counterpart was the Si_3_N_4_ ceramic pin sample with a diameter of 5 mm and a height of 10 mm. *S*_*a*_ was 0.3 ± 0.05 μm. The proportions of its main elements and the important mechanical properties are shown in Tables S1–3. H_2_O_2_ solutions with different concentrations (0%, 10%, 30%, 50%, and 70%) were chosen as the lubricated solution.

### Experimental apparatus and wear tests

All wear tests were conducted on a commercial pin-on-disc friction testing machine (MMW-1 Tribo-tester, Jinan Shidai Shijin Testing Machine Group Co., Ltd., China), as illustrated in Fig. S1. During the tests, the lower AISI 304 stainless steel ring-disc specimen remained stationary while the upper ceramic pin specimen slid against the upper surface of the disc specimen with a rotational motion in a H_2_O_2_ solution, and the remaining surface area was protected with Kapton tape with good oxidation resistance to ensure the accuracy of the measurement results. A three-electrode setup was used for the corrosion measurement of 304 stainless steel during the wear test. The AISI 304 steel samples were used as working electrode and were placed on a surface area of near the luggin capillary. The distance from the measured surface of the disc to the luggin capillary was from 1 mm to 2 mm, as shown in Fig. S1. The calomel electrode was selected as the reference electrode and was put into the luggin capillary. The graphite electrode was selected as the auxiliary electrode. The fixture and oil box were composed of an acrylic material that could not be oxidized in the H_2_O_2_ solution^41^. All electrochemical data were digitally recorded using a Potentiostat Interface 1000 manufactured by Gamry Instruments (Warminster, PA, U.S.A.) connected to a personal computer.

Sliding wear tests on ceramic/ring-disc rubbing pairs were conducted in 0%, 10%, 30%, 50%, and 70% H_2_O_2_ solutions. The rotational speed of the tester was set to 6 rpm. The sliding diameter of the ceramic pin was 60 mm. Therefore, the sliding velocity was 18.84 mm/s. The contact area between the 304 ring-discs and Si_3_N_4_ ceramic pins was 17.6 mm^2^. The nominal load was 100 N, and the calculated test pressure was 5.68 MPa. The test time was 60 min. The wear tests were performed for 10, 20, 30, 40, 50, and 60 min in 30% and 70% H_2_O_2_ solutions, and the wear mass losses every 10 min were obtained. All wear tests were repeated three times under the same conditions to ensure good repeatability of the results. The COFs and CCDs were measured online with a collection frequency of 2 Hz.

### Characterization and analysis

The surface topographies and EDS analysis of the wear surfaces of the 304 ring-discs were examined using a JSM-6701F scanning electron microscope (manufactured by JEOL in Japan). Raman spectra with a resolution of 0.7 cm^−1^ were obtained to identify the metal oxide products. X-ray diffraction patterns were obtained on a Bruker D8 Advance XRD machine to analyse the metal oxide products. The hardness and Young modulus of the metal oxide films at room temperature were measured using a Hysitron TI950 nanoindenter. The surface roughness was measured using laser-interference profilometry (LI–3, Huazhong University of Science and Technology, China). The wear mass losses of the 304 ring-discs were determined by measuring the weights before and after the tests with an analytical balance with a resolution of 0.0000 l g (MS205DU, Shanghai Jiehui Electronic Technology Ltd., China).

## Electronic supplementary material

Supplementary information
