# Supplementary material for: Coupling mechanism between wear and oxidation processes of 304 stainless steel in hydrogen peroxide environments
Source: Sci Rep. 2017 May 24;7:2327. doi: 10.1038/s41598-017-02530-5 (PMC5443796; doi:10.1038/s41598-017-02530-5)
Supplement: Supplementary file 1 — Supplementary information [file 41598_2017_2530_MOESM1_ESM.pdf]

Coupling mechanism between wear and oxidation processes of 304 stainless steel in hydrogen peroxide environments

Conglin Dong[1, 2], Chengqing Yuan[1,*], Xiuqin Bai[1], Jian Li[3], Honglin Qin[4], Xinping Yan[1]

[1]School of Energy and Power Engineering, Wuhan University of Technology, Wuhan 430063, China
[2]State Key Laboratory of Tribology, Tsinghua University, Beijing 100084, China
[3]Wuhan Research Institute of Materials Protection, Wuhan 430030, China.
[4]College of Mechanical and Power Engineering, China Three Gorges University, Yichang, 443002, China
*Corresponding author: ycq@whut.edu.cn (C.Q. Yuan); Fax: +86-27-86549879; Tel: +86-27-86554969.

Supplementary information

Materials preparation. The proportions of main elements of 304 stainless steel are shown in Tables S1. The important mechanical properties of 304 stainless steel ring-disc and Si_3N_4 ceramic pin are shown in Tables S2 and 3 respectively.

Table S1 Chemical compositions (mass %) of the 304 stainless steel specimens.

C	Si	Mn	P	S	Ni	Cr	Fe
0.06	0.42	0.83	0.028	0.005	8.41	18.31	Bal.

Table S2 Mechanical properties of 304 stainless steel

Hardness H/GPa	Modulus E/GPa	Mass density ρ /(g/cm^3)	Tensile strength （MPa）	Yield strength （MPa）	Elongation （%）
7	198	7.85	≥550	≥210	40

Table S3 Mechanical properties of the Si_3N_4 ceramic ball

Hardness H/GPa	Modulus E/MPa	Mass density (g/cm^3)	Bending strength （MPa）	Fracture toughness K_{IC}/MPa m$^{1/2}$	Thermal expansion coefficient (10^{-6} K^{-1})
92	310	3.23	310	9.55	3.2

Experimental apparatus and Wear tests. All wear tests were conducted on a commercial pin-on-disc friction testing machine (MMW-1 Tribo-tester, Jinan Shidai Shijin Testing Machine Group Co., Ltd., China.), as illustrated in Fig. S1. During the tests, the lower AISI 304 stainless steel ring-disc specimen remained stationary while the upper ceramic pin specimen slid against the upper surface of the disc specimen with a rotational motion in a H_2O_2 solution, and the remaining surface area was protected with Kapton tape with good oxidation resistance to ensure the accuracy of the measurement results. A three-electrode setup was used for the corrosion measurement of 304 stainless steel during the wear test. The AISI 304 steel samples were used as working electrode and were placed on a surface area of near the luggin capillary. The distance from the measured surface of the disc to the luggin capillary was from 1 mm to 2 mm, as shown in Fig. S1. The calomel electrode was selected as the reference electrode and was put into the luggin capillary. The graphite electrode was selected as the auxiliary electrode. The fixture and oil box were composed of an acrylic material that could not be oxidized in the H_2O_2 solution. All electrochemical data were digitally recorded using a Potentiostat Interface 1000 manufactured by Gamry Instruments (Warminster, PA, U.S.A.) connected to a personal computer.

The inner diameter, outer diameter, and thickness of the 304 stainless steel ring-discs were 56, 64, and 8 mm, respectively. The counterpart was the Si_3N_4 ceramic pin sample with a diameter of 5 mm and a height of 10 mm. H_2O_2 solutions with different concentrations (0%, 10%, 30%, 50%, and 70%) were chosen as the lubricated media. Sliding wear tests on ceramic/ring-disc rubbing pairs were conducted in 0%, 10%, 30%, 50%, and 70% H_2O_2 solutions. The rotational speed of the tester was set to 6 rpm. The sliding diameter of the ceramic pin was 60 mm. Therefore, the sliding velocity was 18.84 mm/s. The contact area between the 304 ring-discs and Si_3N_4 ceramic pins was 17.6 mm^2. The nominal load was 100 N, and the calculated test pressure was 5.68 MPa. The test time was 60 min. The wear tests were performed for 10, 20, 30, 40, 50, and 60 min in 30% and 70% H_2O_2 solutions, and the wear mass losses every 10 min were obtained. All wear tests were repeated three times under the same conditions to ensure good repeatability of the results. The COFs and CCDs were measured online with a collection frequency of 2 Hz.

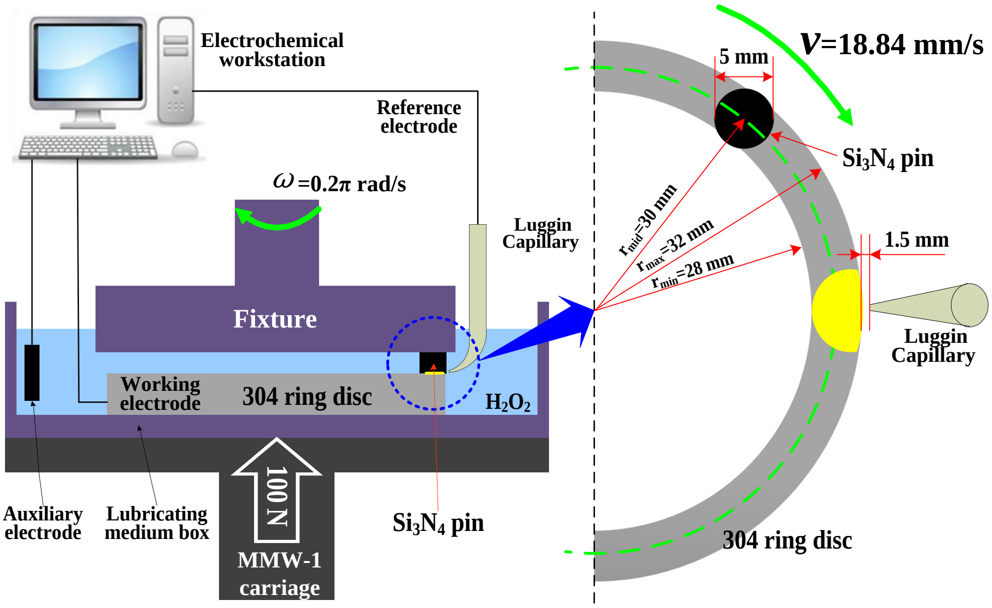

Figure S1. Schematic of the MMW-1 tribo-tester and three-electrode setup used in this study.

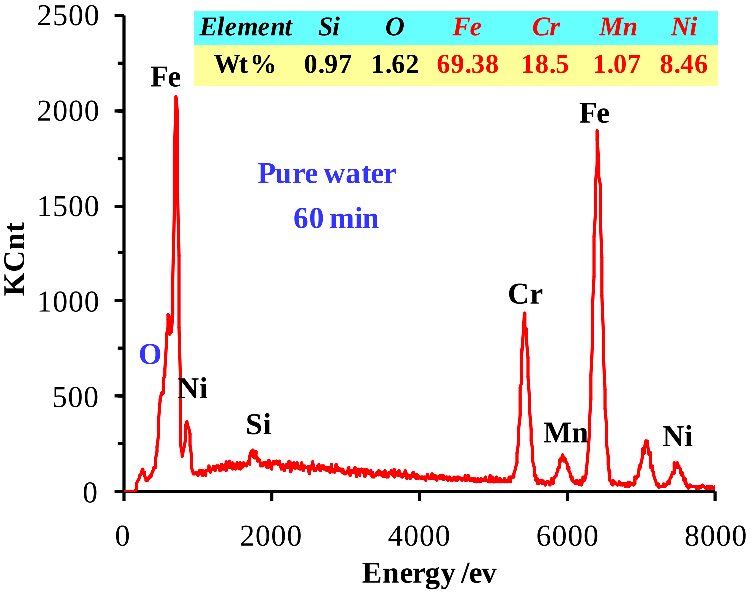

Figure S2. The EDS of Area I in the Fig. 3a
